# Mammography-based screening program: preliminary results from a first 2-year round in a Brazilian region using mobile and fixed units

**DOI:** 10.1186/1472-6874-12-32

**Published:** 2012-10-02

**Authors:** Raphael Luiz Haikel, Edmundo Carvalho Mauad, Thiago Buosi Silva, JacóSaraivadeCastro Mattos, Luciano Fernandes Chala, Adhemar Longatto-Filho, Nestor de Barros

**Affiliations:** 1 Department of Cancer Prevention, Barretos Cancer Hospital, Rua Antenor Duarte Villella, 1331 -, Barretos, São Paulo, SP 14784-400, Brazil; 2 Oswaldo Cruz German Hospital Rua Treze de Maio, 1815 -, Paraíso, São Paulo, SP 01323-903, Brazil; 3 Department of Pathology of Medical School of São Paulo University (LIM14), Av. Dr. Arnaldo, 455 -, Cerqueira César, São Paulo, SP 01246-903, Brazil; 4 Life and Health Sciences Research Institute (ICVS), School of Health Sciences, University of Minho, ICVS/3B’s - PT Government Associate Laboratory, Braga/Guimarães, Portugal; 5 Radiology Institute of Medical School of São Paulo University, Av. Dr. Arnaldo, 455 -, Cerqueira César, São Paulo, SP 01246-903, Brazil; 6 Barretos Cancer Hospital, Rua Antenor Duarte Villella 1331, Bairro Dr. Paulo Prata, Barretos, SP14784-400, Brazil

**Keywords:** Breast cancer, Cancer screening, Cancer prevention, Mammogram

## Abstract

**Background:**

Breast cancer is the most frequently diagnosed cancer and the leading cause of cancer deaths among women worldwide. The use of mobile mammography units to offer screening to women living in remote areas is a rational strategy to increase the number of women examined. This study aimed to evaluate results from the first 2 years of a government-organized mammography screening program implemented with a mobile unit (MU) and a fixed unit (FU) in a rural county in Brazil. The program offered breast cancer screening to women living in Barretos and the surrounding area.

**Methods:**

Based on epidemiologic data, 54 238 women, aged 40 to 69 years, were eligible for breast cancer screening. The study included women examined from April 1, 2003 to March 31, 2005. The chi-square test and Bonferroni correction analyses were used to evaluate the frequencies of tumors and the importance of clinical parameters and tumor characteristics. Significance was set at p < 0.05.

**Results:**

Overall, 17 964 women underwent mammography. This represented 33.1% of eligible women in the area. A mean of 18.6 and 26.3 women per day were examined in the FU and MU, respectively. Seventy six patients were diagnosed with breast cancer (41 (54%) in the MU). This represented 4.2 cases of breast cancer per 1000 examinations. The number of cancers detected was significantly higher in women aged 60 to 69 years than in those aged 50 to 59 years (p < 0.001) or 40 to 49 years (p < 0.001). No difference was observed between women aged 40 to 49 years and those aged 50 to 59 years (p = 0.164). The proportion of tumors in the early (EC 0 and EC I) and advanced (CS III and CS IV) stages of development were 43.4% and 15.8%, respectively.

**Conclusions:**

Preliminary results indicate that this mammography screening program is feasible for implementation in a rural Brazilian territory and favor program continuation.

## Background

Breast cancer is the most frequently diagnosed cancer and the leading cause of cancer deaths among women in both economically developed and developing countries [1]. In 2008, an estimated 1 384 155 cases of breast cancer occurred worldwide, and 458 503 women died from the disease [2]. According to the Brazilian National Institute of Cancer (INCA), 49 240 new cases were expected in the year 2011 [3]. North America, Australia, and some European countries have the highest annual indexes of new cases. The global 5-year survival rates are 73% in developed countries and 57% in developing countries [4].

Breast cancer screening with mammography has proven to be effective in reducing BC mortality in a number of studies, and thus mammography screening has become the standard option worldwide for early detection of tumors [5,6]. Some programs have produced reports of the effective and beneficial use of mobile units (MU) as well as fixed units (FU) for screening, according to specific organizational and geographic situations [7,8].

In regions with easy access to mammography, screening may be conducted in an organized or an opportunistic manner [9]. In opportunistic screening, invitations to screening are passive; here, the individual must decide to undergo screening or they may contact health care providers that offer screening. Thus, opportunistic screening lacks some important requirements for effectiveness, including eligibility parameters, quality assurance of the technical proceedings, follow-up of positive test results, and program evaluation [9]. Most notably, opportunistic screening lacks the population perspective, i.e., the proper setting and organization that is needed to fulfill the aim of mortality reduction in the general population. In contrast, an organized screening program permits recruitment of women in target groups that are difficult to reach, promotes increased return attendance, may decrease health care system costs, and includes routine ongoing quality assurance, evaluation, and overall program monitoring [10]. Organized screening of the general population can increase survival rates to 81% over 5 years [4]. Nevertheless, low participation rates in breast cancer screening remain a concern for many organized programs and have become the focus of intense research [11]. The worldwide rate of participation in the first round of an organized breast screening program ranged from 10.6% in a territory of Canada [12] to 85–95% in Finland [13].

In 2004, the Brazilian guidelines for breast cancer screening with mammography were introduced as a “recommendation”. However, they were not part of a government screening program [14]. Recent convincing evidence has shown that organized screening can effectively reduce breast cancer mortality [7,8,15]. This encouraged us to initiate a screening program in the remote Barretos region of Brazil, and its neighboring areas.

The aim of this study was to evaluate the implementation process and the first 2 years of results from an organized program of breast cancer screening that was introduced in the Barretos region of the state of São Paulo; we also aimed to evaluate the potential impact on public health strategy and cancer control in Brazil.

## Methods

### Participants

The breast cancer screening program was implemented in the Barretos region, São Paulo, and 19 adjacent municipalities. A description of the methodology has previously been published [16]. This study examined results from the first round of the program, which included all cases that had undergone mammography from April 2003 to March 2005 inclusive. The program targeted 54 238 eligible women, aged 40 to 69 years, living in rural and urban areas. Exclusion criteria were a mammographic screening within the previous 2 years and a previous history of breast cancer. The program was initially discussed with community workers and social care assistants from 19 cities in the area. Regular meetings were convened to clarify the goals and steps of the program. This study was authorized by the institutional review board, process number 029/2006.

### Screening units

We used 2 mammography units for screening: one MU and one FU, which was located in the Department of Imaging at the Barretos Cancer Hospital (BCH). Both units had the same mammogram apparatus (Senograph™ 700 T, GE Healthcare, Waukesha, WI, US), with one in the MU and 3 in the FU. Each unit had a capacity for 40 mammograms per day, allowing up to 40 examinations in the MU and 120 examinations in the FU daily.

### Classification report

Single-view mammography was used for the screening and the Breast Imaging-Reporting and Data System (BI-RADS) was used to classify findings in the mammograms [17]. The BI-RADS 1 and BI-RADS 2 classifications were considered a normal mammogram, and no further action was taken. In these screening examinations, the BI-RADS 3 classification was not considered to require further exploration. In contrast, the BI-RADS 4 and BI-RADS 5 classifications required referrals for further examination. Women with carcinoma were treated at the Barretos Cancer Hospital; operated women with no neoplastic lesions were followed according to hospital guidelines. The BCH’s protocol establishes that women with breast changes detected by mammography but with a negative biopsy must be followed up every 6 months for 2 years. If no changes are identified during this time, the mammography is repeated annually.

### Statistical analysis

Statistical analyses were performed using the the chi-square test and Bonferroni correction test, with the level of significance set at p < 0.05. Clinical and pathological data were stored and analyzed with SPSS statistical software (version 17.0, SPSS Inc., Chicago, IL, USA).

### Consent

This project has been approved by the BCH’s Institutional Review Board (process number 029/2006). Written informed consent was obtained from the patient for publication of this report and any accompanying images.

## Results

A total of 17 964 mammograms were performed during the first round of the program; this included 33.1% of all women eligible for screening. Among these, 42.1% (n = 7560) declared they had never had a mammogram before. The average age was 51 years, with a median of 50 years. The MU accounted for 10 521 (58.6%) of the examinations. The FU and MU averaged 18.6 and 26.3 examinations per day, respectively. The results were examined in the following age groups: 40–49 years (48.2%), 50–59 years (34.4%), and 60–69 years (17.4%) (Table 1).

**Table 1:** Characteristics of program participants

**Variable**	**Category**	**n (%)**
Previous MMG	Yes	10,404 (57.9%)
	No	7,560 (42.1%)
Screening unit	Mobile Unit	10,521 (58.6%)
	Fixed Unit	7,443 (41.4%)
Age group (years)	40–49	8,659 (48.2%)
	50–59	6,179 (34.4%)
	60–69	3,126 (17.4%)
Strategies for invitation	Community health agents	8,406 (46.8%)
	Physicians	4,861 (27.1%)
	Radio announcements	2,219 (12.4%)
	Conversations with friends	1,026 (5.7%)
	Other reasons	1,452 (8.1%)
Total		17,964 (100.0%)

Abbreviation: MMG = mammography.

Of the strategies used to motivate adherence to the program, 46.8% of women declared that they were convinced by community healthcare workers, 27.1% were convinced by physicians, 12.4% were motivated by radio announcements, 5.7% were motivated by conversations with friends, and 8.1% were motivated by other reasons (Table 1). A confirmatory examination was necessary for 9.4% (1690/17 964) of the women, and biopsies were obtained from 2.63% (474/17 964). Seventy six cases of breast cancer were identified: 41 (53.9%) of the cases were examined in the MU and 35 (46.1%) in the FU. The positive predictive value (PPV) 1, or the percentage of abnormal examinations that were consistent with cancer, was 4.49% (76/1690); the PPV 2, or the precise diagnosis based on the biopsy, was 16% (76/474).

Of all cancers detected, based on histological analysis, 89.5% were invasive, and carcinoma *in situ* accounted for the remaining 10.5%. Of the invasive cases, 80.9% were invasive ductal carcinoma and 19.1% were lobular invasive carcinomas (Table 2). The frequency of breast cancer in this population was 4.2 cases per 1000 examinations. Cancer frequency was highest in the 60–69-year-old group, with 6.7 cases/1000 examinations; followed by the 40–49-year-old group, with 3.9 cases/1000 examinations; the frequency was lowest in the 50–59-year-old group, with 3.4 cases/1000 examinations (Table 3). The frequency of cancer was significantly higher in women aged 60–69 years compared with the other two groups; 50–59 years (p < 0.001) and 40–49 years (p < 0.001); but no significant difference was found between women aged 40–49 years and 50–59 years (p = 0.164).

**Table 2:** Characteristics of breast cancers

**Variable**	**Category**	**n (%)**
Screening unit	Mobile Unit	41 (53.9%)
	Fixed Unit	35 (46.1%)
Histological type	Invasive ductal carcinoma	55 (72.4%)
	Invasive lobular carcinoma	13 (17.1%)
	Ductal carcinoma *in situ*	8 (10.5%)
Clinical Stage	0-I	33 (43.4%)
	II	31 (40.8%)
	III-IV	12 (15.8%)
Total		76 (100%)

**Table 3:** Cases of cancer according to the number of mammographies by age

**Age group (years)**	**Mammography examinations**	**Cancer cases**	**Cases/1,000 MMG**
40–49	8,666 (48.2%)	34 (44.7%)	3.9
50–59	6,173 (34.4%)	21 (27.6%)	3.4
60–69	3,125 (17.4%)	21 (27.6%)	6.7
Total	17,964 (100%)	76 (100%)	4.2

Based on the Classification of Malignant Tumors (TNM) of the International Union Against Cancer, 43.4% of tumors were diagnosed as early development stage tumors (CS 0 and CS I). This represented 48.6% of tumors detected in the FU and 39.1% of tumors detected in the MU. Advanced stage tumors (CS III and CS IV) accounted for 15.8% of all cases, and represented 14.3% of those detected in the FU and 17% of those detected in the MU (Table 2).

## Discussion

Managers in public health authorities must constantly encourage adherence to breast cancer screening programs to optimize early detection and reduce the associated morbidity and mortality at the population level [18,19]. Participation is naturally a strong prerequisite of the program’s impact; the lack of adherence to the programs can be attributed to a number of factors, including long distances to travel for access to medical facilities [20-22], unavailability of services, low socioeconomic status [21,22], etc. Worldwide, participation in a first round of a breast cancer screening program varies widely, with a range of 10.6% to 54.2% in Canada [12], 34% in Luxembourg [23], 46% in Switzerland [22], 48% in France [24], 47% to 78.1% in Croatia [25], 70.6% in Stockholm [26], 71% in Copenhagen [27], 73% in England [28], 53% to 87% in Spain [29], 75.7% in the Netherlands [30] and 85% to 95% in Finland [13]. The participation rate of women in our program was 33.1% during the first 2-year round. This was lower than the rates reported in some nations [17,31], but it was similar to rates reported for other trials in the initial phases [12,23]. Non-participation in the screening program was partly due to the fact that some of the population had received mammography examinations outside the program. Those women opted to obtain private health insurance, rather than use public national health services [32-34]. The lack of systematic data available on the performance of mammography screening has given rise to deep concern about the actual attendance in screening programs [12,23].

In some European countries, women are identified from the national population registry with a unique personal identification number, and letters of invitation are sent efficiently with an appointment time. Thus, a high attendance rate is guaranteed [13,26,27]. In Luxembourg, the attendance rate in opportunistic screening programs was reduced by adopting a policy of exclusively reimbursing women for mammographs performed in the context of an organized screening program [23]. However, this strategy was not feasible in Brazil, where the constitution guarantees all women the right to receive a mammography free of charge in the public health system.

In Canada, women in the target age range had access to mammography by participating in breast cancer screening programs organized provincially or territorially. The participation rate in seven of these provinces ranged from 10.6% to 54.2%, and five provinces had rates below 30% [12]. In one Canadian survey, 53.7% of women aged 50–69 years self-reported that they had had a screening mammogram within the previous 2 years; it was estimated that 21% of those women were screened through an organized program. The low attendance in organized screening programs may be attributed to: (1) a low capacity for mammography screening; (2) difficulty in shifting from preventive health care practices and opportunistic screening to organized, systematic screening; (3) an inability, in some jurisdictions, to access population information that would facilitate inviting women in the target age range; and (4) a lack of understanding or confidence in the potential benefits of regular mammography screening among women and physicians [12].

In this study, there were several possible explanations for the low participation rate, including: (1) lack of a national registry that stored information on targeted women. The absence of this information may have hampered the ability to identify and access women targeted to participate in the program; (2) approximately 25% of the regional population covered by the breast cancer prevention program had private health insurance, which facilitated access to mammography outside our program; (3) lack of experience in community intervention. This was the first breast cancer screening program with mammography; (5) low adherence of physicians that worked in the municipalities; (6) lack of organization in the Brazilian Health System to accommodate breast cancer screening, and (7) inherent cultural concerns that the examinations may identify cancer and require a referral to and/or examination in a hospital, where they would be exposed to sick people. In the initial stages of this project, the Barretos Cancer Hospital offered mammography and confirmatory exams in the same building that provided the treatments, including chemotherapy, radiotherapy, and surgery. This created a problem due to the close association with confirmed cancer. The problem was resolved in December 2009, with the inauguration of the Barretos Cancer Hospital’s Prevention Department building, where only apparently healthy women were examined.

The number of participants in each age group in the Barretos Cancer Hospital screening program reflected the sizes of the age groups in the eligible population of the Barretos region. The 40 to 49-year-old groups comprised 48.2% of the screened population and 45.2% of the eligible population; the 50 to 59-year-old groups comprised 34.4% of the screened population and 31.9% of the eligible population; and the 60 to 69-year-old groups comprised 17.4% of the screened population and 22.8% of the eligible population. The results from mammogram examinations performed in women aged 40 to 49 years were not significantly different from those performed in women aged 50 to 59. Despite the low number of cases, the results suggested that it would be prudent to initiate mammographic screening in relatively young women. This would optimize the detection of early tumors, despite differences in causality and ethnicity among different regions in Brazil.

This study ratified the large demand for MU examinations; our results corroborated the assumption that the MU was an important tool for facilitating access to mammography [35]. The MU was a critical part of the strategy to overcome obstacles represented by remote areas, poor resources, and/or difficulty in accessing the public health system [35]. A national survey of mammography facilities in the US revealed that 2.4% of mammograms were performed in a MU, but the number of units assessed was low [36]. On the other hand, the Netherlands had 66 units, mostly mobile units, with an annual national coverage of 80% [37]; this provided evidence for the importance and feasibility of MUs.

The MU has been used in developed countries to service population groups with restrictions that prevented participation in screening for breast cancer. These groups generally comprised women with low incomes, low levels of education, and older age, in addition to other limitations, such as residing in a rural area [38-41]. The MU approach has also been used to aid in organized screenings. In small counties, the MU was assumed to provide easy access to a mammogram examination for women who lived at a distance from cities with appropriate resources for breast examinations [42]. Our findings firmly supported this assumption. We found that 10 521 (58.6%) women sought mammograms in the MU, compared with 7443 (41.4%) women that were examined in the FU.

Radiologists in the MU conducted a daily average of 26.3 examinations, with an occupancy rate of 65.7%. In contrast, the FU showed an occupancy rate of 15.5%, with an average of 18.6 examinations per day. Clearly, the units were not operating at full capacity, but the MU in fact answered an underlying need of the population resident in the rural areas more than the FU did with respect to the urban population, which was already partially covered by opportunistic screening outside the program. This highlighted the urgency of implementing more comprehensive communication between the population and public health system to optimize the potential benefits of mammographic screening.

Women were invited to participate in the screening program through various sources, including the media, press, radio, letters, lectures, etc. Some of these methods were previously used in developed countries, such as the invitation letter, which resulted in a participation rate of 85% [43]. Other programs have combined this strategy with media campaigns; these approaches achieved success rates greater than 70% [44]. In our study, the best strategy for promoting participation was an individual suggestion given particularly by community healthcare workers (46.8%). The next most successful strategy was the recommendation from a physician; this persuaded 27.0% of women to have a mammogram. This finding is very important, because a significant percentage of women received information from their doctors.

In the present study, the recall rate of screening was 9.4%, slightly above the 7% recall rate that is most commonly recommended in population-based programs as the upper acceptable limit for the first screening round according to the European Guidelines for Breast Cancer Screening [45]. The Breast Cancer Surveillance Consortium of the U.S. National Cancer Institute, the national breast cancer early detection program of the U.S. Centers for Disease Control and Prevention, and the UK National Health Service Breast Screening Program had recall rates of 13.1%, 11.2%, and 7.4%, respectively, for the first round of screening [46]. The positive predictive value for biopsies was low (16%) compared with the indication from BI-RADS, which ranged from 25% to 40%; this indicated that many patients referred for biopsy had no lesions. This suggested that, although the radiologists had proven experience performing diagnostic mammography, apparently, they did not feel comfortable assessing and diagnosing patients with minimal mammographic alterations. This may have been because they had no specific training in breast radiology; in addition, we used stringent quality assurance and quality control guidelines [47]. The issue of professional training of medical and technical personnel in the assessment units of the screening programs should be among the priorities for consideration in further development of this project.

This program diagnosed 76 cases of breast cancer; the detection rate was 4.23 cases per 1000 examinations. In a large series from UK, a country with higher breast cancer incidence than Brazil, the detection rate ranged from 8.6 to 10.1 cases per 1000 examinations [46]. The absence of a population-based registry in this region of Brazil prevents the determination of breast cancer prevalence in these women for comparison. However, estimates of the INCA show a prevalence of 68.9 cases for 100,000 women in the State of Sao Paulo [48].

One primary objective of screening programs is to identify early stage cancer. We found that 43.4% of tumors were in the early stages of development (EC 0 and EC I). Of note, both symptomatic and asymptomatic women were included in this class, because symptoms were not known initially. This frequency was higher than the 20% reported in the state of Sao Paulo, according to the Oncocentro Foundation of São Paulo [49], and higher than the 14.5% observed in the Barretos region, according to the Cancer Registry of Barretos Cancer Hospital in the period prior to the start of the present screening program. Conversely, in patients with more advanced breast cancer, we observed significant improvements over previous observations, as a result of early screening. We found that tumors in advanced stages (CS III and CS IV) accounted for 15.8% of cancers detected in both units, compared with 39.0% from the state of São Paulo [49] and 40.5% from previous reports of the Barretos region. These findings are important, because they demonstrate the efficiency and relevance of this screening program and they indicate the actual status of breast cancer in Brazil. This approach can lead to new screening programs, or it can be used to improve existing programs, even in the first round. Thus, our findings confirmed, in part, the importance of implementing an organized screening program for the prevention of breast cancer.

Although we obtained accurate data and successfully implemented a system of early detection for breast cancer, the major methodological limitation of this study was that we failed to separate symptomatic from asymptomatic women. This initiative was consistent with the philosophy of the Barretos Cancer Hospital, which included the Department of Prevention. Currently, there is a registry that distinguishes symptomatic from asymptomatic women, but both groups continue to be served by the screening program. In addition, there was a high demand for mammography in the region, which was confirmed by the high number of referrals from doctors for early detection screening. Consequently, the overall problem was large, due to the lack of a health policy priority system for mammographic screening. The early detection program described here was functional and provided acceptable results. The MU approach offers potential for progressive improvement in early detection of breast cancer.

## Conclusions

Our findings support the notion that it would be advisable to plan and set up a comprehensive, population-based breast screening project in the 40–69 age group in our region. This should include the implementation of a monitoring system based on the complete registration of all breast cancer cases in the target population, both symptomatic and asymptomatic, diagnosed both within the screening units and through other diagnostic facilities. Also, high quality, specific training should be required for all professionals involved in the execution, interpretation and diagnostic work-up of screening mammographs.

## Competing interests

The authors have no competing interests to declare.

## Authors’ contributions

RLH, TBS and ALF made substantial contributions to the conception and design of the article, the acquisition, analysis and interpretation of the data, and drafting of the article. ECM, JSCM and NB made substantial contributions to the conception and design of the study. All authors contributed to critical revision of the article for important intellectual content and have given final approval of the version to be published in BMC Women’s Health.

## Pre-publication history

The pre-publication history for this paper can be accessed here:

http://www.biomedcentral.com/1472-6874/12/32/prepub
